# Evaluating a Panel of Autoantibodies Against Tumor-Associated Antigens in Human Osteosarcoma

**DOI:** 10.3389/fgene.2022.872253

**Published:** 2022-04-25

**Authors:** Manli Luo, Songmei Wu, Yan Ma, Hong Liang, Yage Luo, Wentao Gu, Lijuan Fan, Yang Hao, Haiting Li, Linbo Xing

**Affiliations:** ^1^ Luoyang Orthopedic Hospital of Henan Province (Orthopedic Hospital of Henan Province), Luoyang, China; ^2^ Henan Provincial Rehabilitation Hospital, Luoyang, China; ^3^ Henan University of Chinese Medicine, Zhengzhou, China

**Keywords:** osteosarcoma, tumor-associated antigen, autoantibody, detection, early diagnosis, panel

## Abstract

**Background:** The aim of this study was to identify a panel of candidate autoantibodies against tumor-associated antigens in the detection of osteosarcoma (OS) so as to provide a theoretical basis for constructing a non-invasive serological diagnosis method in early immunodiagnosis of OS.

**Methods:** The serological proteome analysis (SERPA) approach was used to select candidate anti-TAA autoantibodies. Then, indirect enzyme-linked immunosorbent assay (ELISA) was used to verify the expression levels of eight candidate autoantibodies in the serum of 51 OS cases, 28 osteochondroma (OC), and 51 normal human sera (NHS). The rank-sum test was used to compare the content of eight autoantibodies in the sera of three groups. The diagnostic value of each indicator for OS was analyzed by an ROC curve. Differential autoantibodies between OS and NHS were screened. Then, a binary logistic regression model was used to establish a prediction logistical regression model.

**Results:** Through ELISA, the expression levels of seven autoantibodies (ENO1, GAPDH, HSP27, HSP60, PDLIM1, STMN1, and TPI1) in OS patients were identified higher than those in healthy patients (*p* < 0.05). By establishing a binary logistic regression predictive model, the optimal panel including three anti-TAAs (ENO1, GAPDH, and TPI1) autoantibodies was screened out. The sensitivity, specificity, Youden index, accuracy, and AUC of diagnosis of OS were 70.59%, 86.27%, 0.5686, 78.43%, and 0.798, respectively.

**Conclusion:** The results proved that through establishing a predictive model, an optimal panel of autoantibodies could help detect OS from OC or NHS at an early stage, which could be used as a promising and powerful tool in clinical practice.

## Introduction

Osteosarcoma (OS) is one of the most common primary malignancies of bone sarcomas, whose incidence rate is less than 1% of all tumors in the United States (Howlader et al., 2020; Siegel et al., 2020). Worldwide, OS occurs in about 1–3 cases per million people annually (Kansara et al., 2014). OS has a bimodal age distribution and occurs mostly in children and adolescents; another type occurs in people over 60 years old (Corre et al., 2020). In addition, once clinically diagnosed with OS, there are about 15–20% patients who have detectable metastases, with a 5-year survival rate of ∼20% (Wang et al., 2020). Surgical resection combination with chemotherapy is one of the common measures to treat the tumor (Ta et al., 2009). As OS is highly malignant and has low incidence and non-specific initial symptoms, it is often misdiagnosed or neglected. However, until now, there are no efficient biomarkers for detection in clinics. Thus, in order to treat the patients timely, it is essential to diagnose OS at an early stage with a non-invasive method, which has high specificity/sensitivity.

Gene mutations can cause the changes in the gene and abnormal expression of gene products, resulting in the occurrence and development of tumors (Sparano et al., 2013). These abnormal proteins often appear along with the development of the tumor, some of which can appear in the blood circulation system of the body which are called tumor-associated antigens (TAAs) (Tan and Zhang, 2008; Restifo et al., 2012). These TAAs can be recognized by the immune system of tumor patients, and then, anti-TAA autoantibodies were produced. An important fact is that autoantibodies can exist stably in the host for even months or years before clinical diagnosis (Lu et al., 2008). Meanwhile, the content of autoantibodies in the sera of normal humans is low, while in tumor patients it is much higher, so these autoantibodies have great potential as tumor biomarkers in the early immunodiagnosis of OS (Tan and Zhang, 2008; Bracci et al., 2012; Anderson et al., 2015). It was known to all that the anti-p53 autoantibody was used for diagnosing different tumors (Soussi, 2000). Various studies have illustrated the role of autoantibodies in diagnosing tumors, such as in hepatocellular carcinoma (Zhang and Tan, 2010), lung cancer (Huo et al., 2020), breast cancer (Qiu et al., 2018), and esophageal cancer (Pan et al., 2019). However, the function of a single autoantibody was limited; many studies have proven that a custom-made panel of autoantibodies could enhance the diagnostic performance of tumors (Chen et al., 2020; Huang et al., 2020; Okada et al., 2020).

Serological proteome analysis (SERPA) is one of the methods to screen new TAAs. In our study, new autoantibodies were discovered by SERPA, the method which was mature in our laboratory (Dai et al., 2017). Its main advantage is that it can directly screen the proteins extracted from tumor tissues or cells as antigen sources. As enzyme-linked immunosorbent assay (ELISA) is rapid, simple, and inexpensive, it becomes the most commonly used method to detect TAAs or autoantibodies as serum tumor biomarkers.

Our previous study (Li et al., 2021) has found a panel of eight candidate serum autoantibodies with SERPA. In this study, these eight candidate autoantibodies were detected by indirect ELISA. Differentially expressed autoantibodies were screened out among eight candidate autoantibodies. Then, an optimal panel of autoantibodies was established by the logistic regression statistical method. Eventually, the diagnostic value of the panel was evaluated in the detection of OS in subgroups.

## Methods and Materials

### Study Population

There were 51 clinically confirmed OS cases, 28 osteochondroma (OC), and 51 NHS included in this study, which were matched by gender and age. All healthy samples were confirmed with no malignant diseases. The sera of 51 OS cases and 28 benign controls (OC) were obtained from the Luoyang Orthopedic Hospital of Henan Province (Orthopedic Hospital of Henan Province) from November 2013 to June 2016, and the healthy control of 51 cases was obtained from healthy people who were examined in the physical examination department at the same hospital in the same period. All blood samples were centrifuged at 3,000 rpm for 10 min after collection and then stored in a refrigerator at −80°C before the experiment. All subjects participating in the study have signed the informed consent form. The study had been approved by the Ethics Committee of Luoyang Orthopedic Hospital of Henan Province.

### Serological Proteome Analysis

Two osteosarcoma cell lines U2-OS and Saos-2 were cultured. Specific experimental methods and results were based on our published literature (Li et al., 2021).

### Enzyme-Linked Immunosorbent Assay

Eight recombinant proteins including ENO1, GAPDH, HSP27, HSP60, NPM1, PDLIM1, STMN1, and TPI1 were used as antigens to detect the corresponding autoantibodies. Purified ENO1 protein was bought from Sigma. GAPDH (ab77109), TPI1 (ab100826), HSP60 (ab78430), PDLIM1 (ab177676), and STMN1 (ab87492) were purchased from Abcam Inc (Cambridge, MA, United States). NPM1 was provided by the Henan Key Laboratory of Tumor Epidemiology.

Autoantibodies were detected by indirect ELISA. Each purified protein was diluted to an appropriate concentration with a coating solution (ENO1, GAPDH, HSP27, HSP60, NPM1, PDLIM1, STMN1, and TPI1 to final concentrations of 1.0 μg/ml). For the assay, 100 μl of the coating solution was added to each well of a 96-well ELISA plate. Then, the plate was sealed with a plastic wrap and placed in the refrigerator at 4°C for 16 h. After discarding the coating solution, 200 μl of 2% BSA was added into each well and was then placed in the refrigerator at 4°C overnight for 16 h. The plate was washed with 1 × PBST solution three times on the 96-well automatic washer. Then, 100 μl of pre-diluted serum samples diluted at 1:100 in 1% BSA in the deep well plate was added for incubating at 37°C for 1 h. After being washed with the 1 × PBST solution five times, the plates were incubated with secondary antibody, horseradish peroxidase (HRP)-conjugated goat anti-human IgG (H+L), diluted by 1:4,000 in 1% BSA at 37°C for 1 h. After also being washed with 1 × PBST solution five times, the substrate (1 mg/ml 2,2-azino-bis [3-ethylbenzthiazoline-6-sulfonic acid] with 0.005% hydrogen peroxide in citrate buffer, pH 4.6) was used as detecting reagents. The optical density (OD) was measured at 405 nm using an automated plate reader. All serum samples were assayed in duplicate.

### Statistical Analysis

All statistical analyses were performed by SPSS 21.0 and GraphPad Prism 6.0 software. The Mann–Whitney U test was used to compare the expression level of autoantibodies if there were any abnormal distribution (Kolmogorov–Smirnov test). Receiver operating characteristic (ROC) curves were conducted to evaluate the diagnostic value of each autoantibody and the prediction model. The binary backward stepwise logistic regression prediction model (condition) was used to select the optimal panel of autoantibodies. The comparison between two AUCs was conducted by Medcalc 11 software. The predicting probability *p* = 0.5 was set as the cutoff point. *p* was two-tailed and less than 0.05 was considered significant.

## Results

### Basic Clinical Characteristics

There were 51 new cases of OS, 28 OC, and 51 healthy people included in this study. The basic clinical characteristics are shown in Table 1. There was no statistically significant difference in gender and age between OS and NHS (*p* > 0.05).

**TABLE 1:** Basic clinical characteristics of OS, OC, and NHS.

	OS	OC	NHS
	*n* = 51	*n* = 28	*n* = 51
Age			
Mean, SD	24.88, 14.29	17.21, 12.02	26.35, 13.40
Range	4–64	4–52	5–59
Gender (%)			
Male	33 (64.71)	19 (67.86)	33 (64.71)
Female	18 (35.29)	9 (32.14)	18 (35.29)
TNM (%)			
I	6 (11.76)		
IIA	23 (45.10)		
IIB	9 (17.65)		
IV	9 (17.65)		
Unknown	4 (7.84)		
Degree of differentiation (%)			
1–2	4 (7.84)		
3–4	41 (80.39)		
Unknown	6 (11.76)		
Tumor size			
<5 cm	13 (25.49)		
≥5 cm	38 (74.51)		

### Expression of Candidate Autoantibodies in Three Groups

**FIGURE 1:**
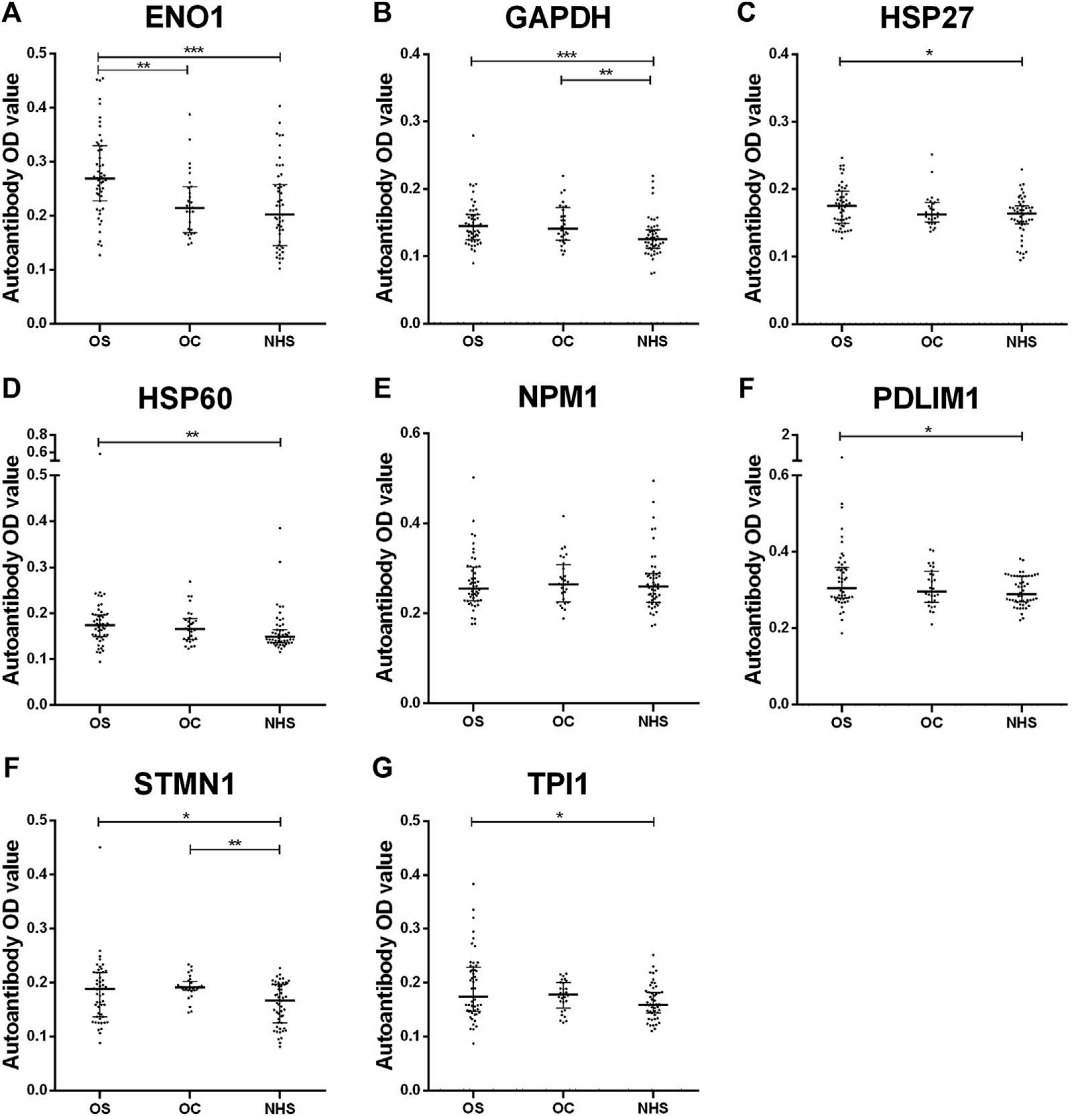
Scatter plots of serum levels of autoantibodies against eight TAAs in OS, OC and NHS (median with interquartile). *p* < 0.001; *p* < 0.01; and *p* < 0.05. OS, osteosarcoma; OC, osteochondroma; NHS, normal human sera.

Based on our previous study, using SERPA, eight TAAs were screened out. Here, by ELISA, seven autoantibodies whose OD values in OS were higher than those in NHS and *p*-values less than 0.05 were screened out (ENO1, GAPDH, HSP27, HSP60, PDLIM1, STMN1, and TPI1). More details are shown in Figure 1 and Supplementary Table S1.

### Expression of Eight Autoantibodies in Osteosarcoma and Control Group

**FIGURE 2:**
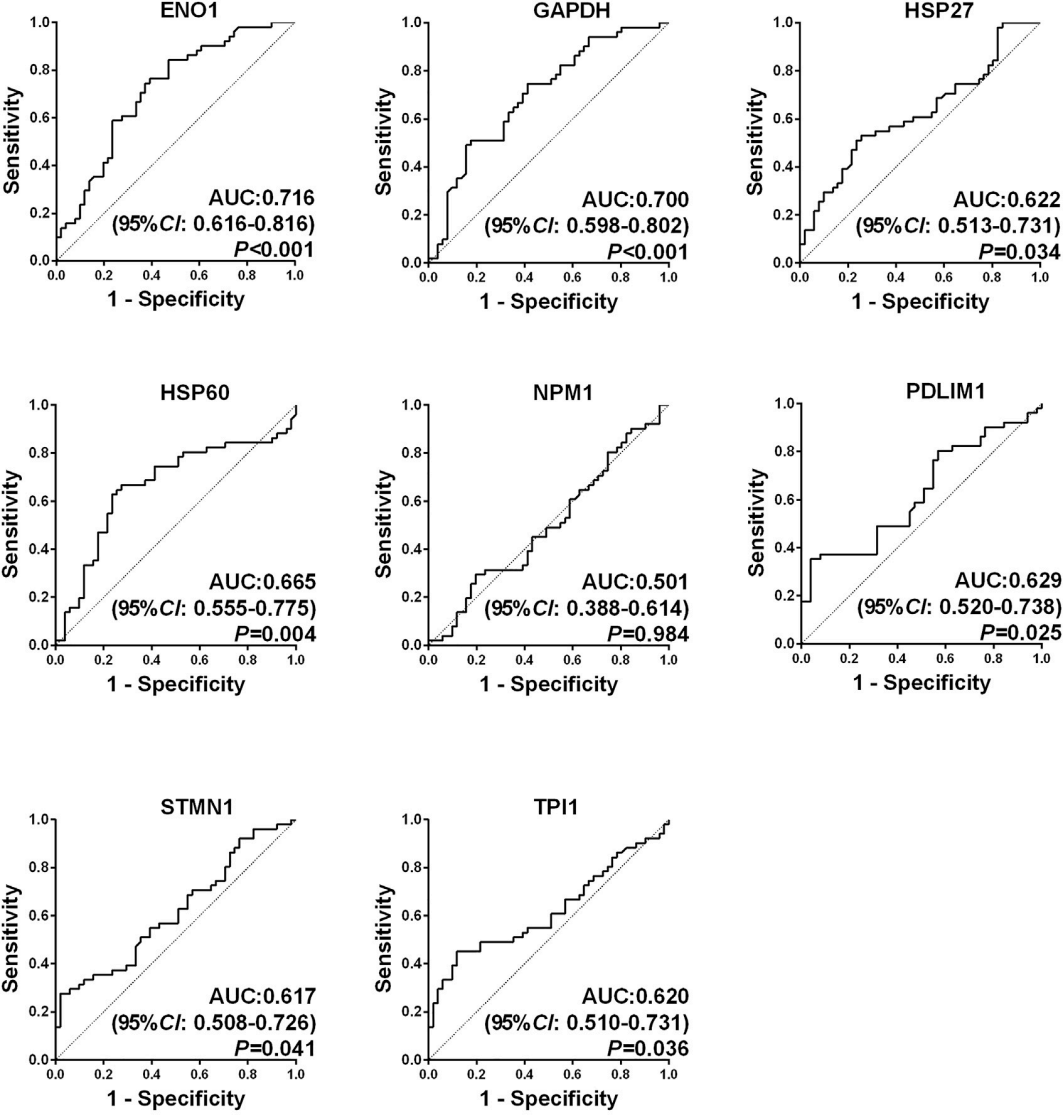
Receiver operating characteristic (ROC) curves of eight candidate tumor-associated autoantibodies (TAAbs).

ROC curves of eight autoantibodies in the sera of OS and NHS were measured, as shown in Figure 2. The contents of ENO1, GAPDH, HSP27, HSP60, PDLIM1, STMN1, and TPI1 autoantibodies in the sera of OS were higher than those of NHS, and the AUC was statistically significant (*p* < 0.05). The range of the AUC of seven autoantibodies was 0.617–0.716. Then, these seven TAAs were further included in the next stage for verification.

### The Optimal Panel of Autoantibodies in Diagnosing Osteosarcoma Was Established

**FIGURE 3:**
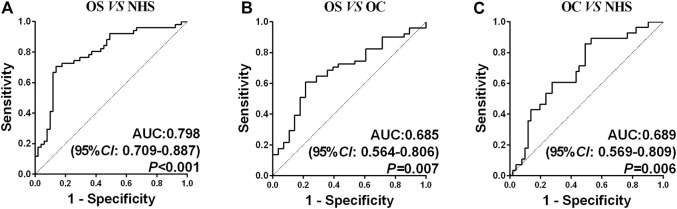
ROC curve analysis of the prediction model with the TAA panel of OS detection. **(A)** Prediction model with three TAAs for OS detection in healthy controls. **(B)** Prediction model with three TAAs for OS detection in OC. **(C)** Prediction model with three TAAs for OC detection in healthy controls.

A backward stepwise (condition) logistic regression predictive model was conducted to select the optimal model among seven biomarkers in diagnosing OS. The predictive probability in diagnosing OS is as follows: PRE (*P*= OS, 3 TAAs) = 1/(1 + EXP (−(−6.679 + 9.686 × ENO1 + 17.286 × GAPDH +11.178 × TPI1))). Predicting probability *p* = 0.5 was set as the cutoff point. The sensitivity, specificity, Youden index, accuracy, and AUC of this model were 70.59%, 86.27%, 0.5686, 78.43%, and 0.798, respectively. The ROC curves among three groups are shown in Figure 3. The AUC was statistically significant (*p* < 0.05).

### The Diagnostic Value of the Optimal Panel in the Early Stage and Ages Up to 19 Years Old

The subjects were divided into two subgroups as follows: early stage (I–II) and late stage (III–IV). In both stages, the AUC of OS was higher than that of OC or NHS (*p* < 0.05), except for the OS (III–IV) vs. OC (*p* > 0.05). When OS was divided by 19 years old, in these two age subgroups, the AUC of OS was higher than that of OC or NHS (*p* < 0.05). More details are shown in Supplementary Figures S1, S2 and Supplementary Table S2. In addition, either in stage groups or age groups, the comparison between two subgroups (neither vs. OC or NHS) had statistical significance.

## Discussion

As OS has a high rate of amputation, disability, and death, it is important to detect and treat OS at an early stage (Wafa and Grimer, 2006). The non-invasive serological screening method is optimal. Autoantibodies as an autoimmune phenomenon, which have been reported in many studies, can be served as an effective means (Qin et al., 2019; Huo et al., 2020; Okada et al., 2020; Qiu et al., 2020). SERPA is a promising approach used in identifying autoantibodies (Dai et al., 2016). In the previous study, eight candidate TAAs which had statistical significance between OS and NHS were selected for further validation based on SERPA. In this study, we measured the contents of ENO1, GAPDH, HSP27, HSP60, NPM1, PDLIM1, STMN1, and TPI1 autoantibodies by ELISA. The expression levels of seven autoantibodies were higher in OS than those in NHS. However, a single biomarker was limited in diagnosing OS. Based on these data, we established a predictive model containing three biomarkers including ENO1, GAPDH, and TPI1 to help improve the diagnostic performance. Afterward, the diagnostic value was evaluated among OS, OC, and NHS.

These three TAAs have been studied in many tumors. The expression of ENO1 in the invasion and metastasis of tumor cells was concerned by many studies with high expressions in OS (Chen et al., 2014), breast cancer (Gao et al., 2013), lung cancer (Zhang et al., 2018), and hepatocellular carcinoma (Zhu et al., 2018; Deng et al., 2020). During cancer development and progression, glycolysis and glyceraldehyde-3-phosphate dehydrogenase (GAPDH) played an important role, such as in colorectal adenocarcinoma (Brzozowa-Zasada et al., 2018) and breast cancer (Goes et al., 2010). Ogino et al. (2007) had proven that GAPDH mRNA was associated with lung metastasis in osteosarcoma. The poor prognosis of peripheral T-cell lymphoma (Ludvigsen et al., 2018) or hepatocellular carcinoma (Jiang et al., 2017) was associated with triosephosphate isomerase (TPI1). Research studies using TPI1 in early diagnosis of OS was hardly performed.

Many studies have shown that the diagnostic ability of a single biomarker was limited, and the combination of several ones could increase the diagnostic performance in different cancers (Zhang et al., 2016; Li et al., 2017; Wang S et al., 2018). For OS, Gao et al. (2020) reported a meta-analysis that the combination of several circulating miRNAs had a high accuracy whose sensitivity, specificity, and AUC were 0.79, 0.89, and 0.90. Three lncRNAs (RP1-261G23.7, RP11-69E11.4, and SATB2-AS1) were selected as independent prognostic factors for OS patients (Ying et al., 2020). Nevertheless, the use of a panel of autoantibodies was proven in other kinds of tumors but not in OS. Wang et al. (2019) found an optimal panel of nine autoantibodies (RalA, p62, p53, koc, p90, p16, c-myc, AHSG, and 14-3-3zeta), whose sensitivity and specificity can reach 61.4% and 85.0%, respectively. Furthermore, combined with CA125 used in clinical practice, the sensitivity, specificity, and AUC could reach 94.7, 78.2, and 0.914%. Zhang et al. (2018) selected a panel of four autoantibodies, which provided a higher diagnostic performance with AUC 0.838 in the training cohort and 0.872 in the validation cohort for ESCC detection. In hepatocellular carcinoma, six autoantibodies (Sui1, p62, RalA, p53, NY-ESO-1, and c-myc) may have both diagnostic and prognostic values in HCC (Okada et al., 2020).

Until now, studies focusing on using autoantibodies as biomarkers in early diagnosis of OS are almost rare. Our research is leading in this area. In this study, the range of the AUC of a single autoantibody was 0.617–0.716, which was difficult to meet the requirements for early diagnosis in clinical practice. By establishing the logistic regression predictive model, which was widely used to classify diseases, especially in cancers (Wang S et al., 2018; Cui et al., 2021), it proved to be a conventional analytical method. This model contained three biomarkers finally, and the sensitivity, specificity, Youden index, accuracy, and AUC of this model were 70.59%, 86.27%, 0.5686, 78.43%, and 0.798, respectively, between OS and NHS. By comparing the OS and OC, the sensitivity, specificity, Youden index, accuracy, and AUC of this model were 70.59%, 57.14%, 0.2773, 65.82%, and 0.685, respectively, and the comparison between these two AUCs was not statistically significant. The panel could help identify OS patients from neither benign tumor nor normal people, which means this panel could serve as good diagnostic biomarkers. Based on these aforementioned studies, we were confident in using the panel of autoantibodies to detect OS at an early stage; thus, the disease could be treated earlier. In addition, if the panel could be combined with common clinical indicators in the future, the results are hopeful.

In this study, OS and NHS patients were matched by age and gender. After the predictive model was established, the predictive value was calculated in benign tumors as well. It was reported that serum soluble B7-H3 concentrations were significantly higher than those in benign diseases and healthy people (*p* < 0.05) (Wang L et al., 2018). Zhao et al. (2017) presented the result that the expression of HBME-1 was higher in OS tissues than that in OC and normal bone tissues. The AUC of the HBME-1 expression was 0.864, with a sensitivity of 80.92%, specificity of 91.89%, and an accuracy of 84.51%. Xu et al. (2010) reported that the basic fibroblast growth factor (b-FGF) and endostatin were expressed higher in OS than in OC tissue. However, they were rarely focused on evaluating serological diagnostic biomarkers of OS in this field, which was one of the advantages of this study. In addition, this study did further research works on the diagnostic value of the model on the early (I–II) and late stages (III–IV), and the subgroup was divided as the subjects of age 19. In the early stage (I–II), the sensitivity, specificity, Youden index, accuracy, and AUC of diagnosis of OS were 68.42%, 86.27%, 0.5470, 78.65%, and 0.796, between OS and NHS. This indicated that this panel could be a promising and powerful tool in clinical practice, especially for people at the early stage of OS, which could help treat these patients early. While in the subgroup of age, the comparison of the AUC between these two groups did not have statistical significance.

At present, autoantibodies against tumor-associated antigens were studied by many researchers in the field of cancer for early diagnosis or prognosis. In our study, eight candidate tumor-associated antigens’ autoantibodies were screened out through the SERPA technology, a sufficient screening method. On the other hand, this study included benign controls, which confirmed the results. However, there still existed some limitations. At first, the number of the subjects was limited as OS was difficult to collect, as well as the inequality of each stage. It could be better if a larger number of OS and OC samples could be collected in the future. Also, if it could be combined with other types of biomarkers, it would be better for clinical practice.

## Conclusion

In conclusion, our results have proven that using the binary logistic regression predictive model, an optimal panel of autoantibodies could help detect OS from OC or NHS at an early stage as a promising and powerful tool in clinical practice.

## Data Availability

The original contributions presented in the study are included in the article/Supplementary Material, and further inquiries can be directed to the corresponding authors.
