# Supplementary material for: Evaluating a Panel of Autoantibodies Against Tumor-Associated Antigens in Human Osteosarcoma
Source: Front Genet. 2022 Apr 25;13:872253. doi: 10.3389/fgene.2022.872253 (PMC9081566; doi:10.3389/fgene.2022.872253)
Supplement: Supplementary file 1 [file DataSheet1.docx]

Supplementary Material

# Supplementary Tables

| **Supplementary Table 1.** OD of autoantibodies of OS, OC and NHS (Median (*P25*~*P75*)). | | | | | | | | | |
| --- | --- | --- | --- | --- | --- | --- | --- | --- | --- |
| Anti-TAA autoantibody | OS (OD) | OC (OD) | NHS (OD) | *Z** | *P** | *Z^#^* | *P^#^* | *Z^&^* | *P^&^* |
| ENO1 | 0.269(0.228~0.329) | 0.215(0.170~0.254) | 0.202(0.145~0.258) | -3.758 | <0.001 | -3.177 | 0.001 | -0.610 | 0.542 |
| GAPDH | 0.145 (0.125~0.162) | 0.141 (0.123~0.172) | 0.125 (0.112~0.139) | -3.477 | 0.001 | -0.056 | 0.955 | -2.670 | 0.008 |
| HSP27 | 0.175 (0.149~0.197) | 0.162 (0.151~0.180) | 0.164 (0.148~0.175) | -2.125 | 0.034 | -1.071 | 0.284 | -0.507 | 0.612 |
| HSP60 | 0.174 (0.149~0.197) | 0.165 (0.143~0.188) | 0.149 (0.136~0.164) | -2.871 | 0.004 | -0.682 | 0.496 | -1.819 | 0.069 |
| NPM1 | 0.255 (0.228~0.303) | 0.265 (0.226~0.308) | 0.260 (0.225~0.289) | -0.020 | 0.984 | -0.492 | 0.623 | -0.405 | 0.686 |
| PDLIM1 | 0.305 (0.279~0.358) | 0.297 (0.268~0.348) | 0.288 (0.268~0.336) | -2.249 | 0.025 | -0.948 | 0.343 | -0.830 | 0.406 |
| STMN1 | 0.188 (0.137~0.219) | 0.191 (0.186~0.202) | 0.167 (0.126~0.197) | -2.041 | 0.041 | -1.138 | 0.255 | -2.952 | 0.003 |
| TPI1 | 0.174 (0.146~0.229) | 0.178 (0.153~0.200) | 0.159 (0.143~0.183) | -2.095 | 0.036 | -0.810 | 0.418 | -1.783 | 0.075 |

OD: optical density. *P25*: lower quartile, *P75*: upper quartile*.* *OS *vs* NHS; ^#^OS *vs* OC; ^&^OC *vs* NHS.

| **Supplementary Table 2.** Diagnostic value of the optimal panel for different stages and ages. | | | | | | | | | | | | | | |
| --- | --- | --- | --- | --- | --- | --- | --- | --- | --- | --- | --- | --- | --- | --- |
| Group | n | Positive (%) | AUC | *95%CI* | *P* | Sensitivity  (%) | Specificity  (%) | YI | +LR | -LR | *Kappa* | PPV  (%) | NPV  (%) | Accuracy  (%) |
| Early-stage  (I-II) | 38 | 26 (68.42) | 0.796^*^ | 0.701-0.891 | <0.001 | 68.42 | 86.27 | 0.5470 | 3.71 | 0.27 | 0.56 | 78.79 | 78.57 | 78.65 |
|  |  |  | 0.675^#^ | 0.544-0.806 | 0.016 | 68.42 | 57.14 | 0.2556 | 2.17 | 0.75 | 0.26 | 68.42 | 57.14 | 63.64 |
| Late-stage  (III-IV) | 9 | 6 (66.67) | 0.767^*^ | 0.567-0.967 | 0.011 | 66.67 | 86.27 | 0.5294 | 0.86 | 0.07 | 0.45 | 46.15 | 93.62 | 83.33 |
|  |  |  | 0.691^#^ | 0.461-0.920 | 0.089 | 66.67 | 57.14 | 0.2381 | 0.50 | 0.19 | 0.18 | 33.33 | 84.21 | 59.46 |
| Age up to 19 years old | 25 | 17 (68.00) | 0.775^*^ | 0.661-0.889 | <0.001 | 68.00 | 86.27 | 0.5427 | 2.42 | 0.18 | 0.55 | 70.83 | 84.62 | 80.26 |
|  |  |  | 0.661^#^ | 0.511-0.812 | 0.024 | 68.00 | 57.14 | 0.2514 | 1.42 | 0.50 | 0.25 | 58.62 | 66.67 | 62.26 |
| Age over 19 years old | 26 | 19 (73.08) | 0.820^*^ | 0.716-0.923 | <0.001 | 73.08 | 86.27 | 0.5935 | 2.71 | 0.16 | 0.59 | 73.08 | 86.27 | 81.82 |
|  |  |  | 0.707^#^ | 0.566-0.849 | 0.017 | 73.08 | 57.14 | 0.3022 | 1.58 | 0.44 | 0.30 | 61.29 | 69.57 | 64.81 |
| OS | 51 | 36 (70.59) | 0.798^*^ | 0.709-0.887 | <0.001 | 70.59 | 86.27 | 0.5686 | 5.14 | 0.34 | 0.57 | 83.72 | 74.58 | 78.43 |
| OC | 28 | 12 (42.86) | 0.685^#^ | 0.564-0.806 | 0.017 | 70.59 | 57.14 | 0.2773 | 3.00 | 0.94 | 0.27 | 75.00 | 51.61 | 65.82 |
| NHS | 51 | 7 (13.73) |  |  |  |  |  |  |  |  |  |  |  |  |

* comparison between OS and NHS; ^#^ comparison between OS and OC.

# Supplementary Figures

#
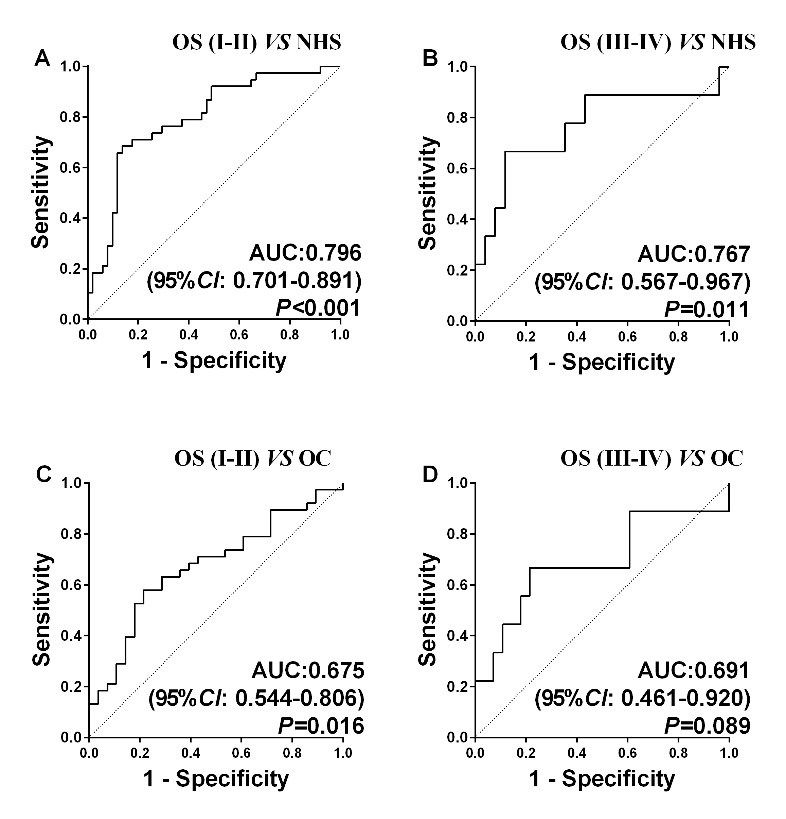


**Supplementary Figure 1.** ROC curves analysis of prediction model with the TAA panel in early and late stage of OS detection. **(A)** the prediction model with 3 TAAs for early-stage OS detection in healthy controls. **(B)** the prediction model with 3 TAAs for late-stage OS detection in healthy controls. **(C)** the prediction model with 3 TAAs for early-stage OS detection in OC. **(D)** the prediction model with 3 TAAs for late-stage OS detection in OC.





**Supplementary Figure 2.** ROC curves analysis of prediction model with the TAA panel in different age of OS detection. **(A)** the prediction model with 3 TAAs for OS (up to 19 years old) detection in healthy controls. **(B)** the prediction model with 3 TAAs for OS (over 19 years old) detection in healthy controls. **(C)** the prediction model with 3 TAAs for OS (up to 19 years old) detection in OC. **(D)** the prediction model with 3 TAAs for OS (over 19 years old) detection in OC.
